# Cardiovascular autonomic dysfunction in “Long COVID”: pathophysiology, heart rate variability, and inflammatory markers

**DOI:** 10.3389/fcvm.2023.1256512

**Published:** 2023-09-01

**Authors:** Karina Carvalho Marques, Juarez Antônio Simões Quaresma, Luiz Fábio Magno Falcão

**Affiliations:** ^1^ Center for Biological Health Sciences, State University of Pará (UEPA), Belém, Brazil; ^2^ School of Medicine, São Paulo University (USP), São Paulo, Brazil; ^3^ Tropical Medicine Center, Federal University of Pará (UFPA), Belém, Brazil

**Keywords:** Long COVID, autonomic nervous system, autonomic diseases, pathophysiology, inflammation, heart rate variability

## Abstract

Long COVID is characterized by persistent signs and symptoms that continue or develop for more than 4 weeks after acute COVID-19 infection. Patients with Long COVID experience a cardiovascular autonomic imbalance known as dysautonomia. However, the underlying autonomic pathophysiological mechanisms behind this remain unclear. Current hypotheses include neurotropism, cytokine storms, and inflammatory persistence. Certain immunological factors indicate autoimmune dysfunction, which can be used to identify patients at a higher risk of Long COVID. Heart rate variability can indicate autonomic imbalances in individuals suffering from Long COVID, and measurement is a non-invasive and low-cost method for assessing cardiovascular autonomic modulation. Additionally, biochemical inflammatory markers are used for diagnosing and monitoring Long COVID. These inflammatory markers can be used to improve the understanding of the mechanisms driving the inflammatory response and its effects on the sympathetic and parasympathetic pathways of the autonomic nervous system. Autonomic imbalances in patients with Long COVID may result in lower heart rate variability, impaired vagal activity, and substantial sympathovagal imbalance. New research on this subject must be encouraged to enhance the understanding of the long-term risks that cardiovascular autonomic imbalances can cause in individuals with Long COVID.

## 1. Introduction

Long COVID Syndrome, know as Long COVID, can be described as the persistence signs and symptoms that continue or develop for more than 4 weeks after acute COVID-19 infection (1). During clinical evaluation, differential diagnosis and the identification of associated pathologies unrelated to SARS-CoV-2 infection are essential (2). Manifestations of Long COVID occur in approximately 50%–80% of previously symptomatic patients with COVID-19 who have recovered (3). Cardiovascular autonomic dysfunction or dysautonomia involves inadequate autonomic nervous system (ANS) function, resulting in various cardiovascular symptoms. Dysautonomia can be acute, chronic, progressive, irreversible, or variable and can accompany infectious or non-infectious diseases. Cardiovascular autonomic dysfunction can be assessed by measuring heart rate variability (HRV) using linear (time or frequency domain) and non-linear methods (4). HRV measures RR interval variation, autonomic function and neurocardiac (5) from simple bedside analysis to more sophisticated markers (6), in patients with cardiac or non-cardiac diseases (7) with analysis of vagal function (8). The cardiovascular autonomic pathophysiology of Long COVID is unclear, but neurotropism, hypoxia, or viral-mediated pathways may be associated with this condition. The symptoms of cardiovascular autonomic dysfunction include orthostatic intolerance, chest pain, palpitations, reduced exercise tolerance, and “brain fog” (9, 10).

According to Raveendran et al. (2021) (10), the symptoms of Long COVID occur due to chronic inflammatory persistence or immune responses related to antibody production. Individuals with altered immunity or those who are reinfected may develop viraemia (11). To better understand Long COVID, investigating whether elevated levels of inflammatory markers are present long-term is necessary, potentially improving the prognostic stratification of this disease (12–16).

Autonomic tests can be used to analyse the possible causal relationships of dysautonomia (17) in individuals with Long COVID. Associations between HRV and various inflammatory markers appear to alter cardiovascular autonomic modulation. HRV monitoring may also result in better disease stratification in patients with Long COVID. The objective of this review was to provide information on long-term cardiovascular autonomic dysfunction in patients with Long COVID and its impact on morbidity and mortality in this patient population.

## 2. Autonomic pathophysiology of long COVID

The persistent neurological complications observed in Long COVID likely result from damage to the central nervous system (CNS) and peripheral nervous system (PNS). This may involve a complex pathophysiology, including direct viral neuronal damage, neuroinflammation, disruption of the blood-brain barrier (BBB), microvasculitis, and hypoxia (18). Through the angiotensin-converting enzyme 2 (ACE2), SARS-CoV-2 (the causative agent of COVID-19) binds to nervous system (NS) cells in the brain, the choroid plexus, and the ventral posterior nucleus of the thalamus. SARS-CoV-2 also binds to α7 nicotinic acetylcholine receptors (α7 nAChRs) (19). Limited data are available on the SARS-CoV-2 neuroinvasive routes and NS infection (20); however, neuroinflammation with substantial immune infiltration has been observed (21).

After direct infection by SARS-CoV-2, neurotropic effects play a considerable role in the pathogenesis of Long COVID. SARS-CoV-2 may use ACE2 to enter CNS and PNS cells through haematogenous or transsynaptic pathways (22). Although the neurotropic pathways are unclear, the virus is likely to cross the BBB or directly or indirectly damage it. The SARS-CoV-2 spike protein (S12) can also damage the integrity of the BBB, either independently or in conjunction with other cell mediators (23). Indirect damage occurs through endothelial cells and pericytes or activation of the autoimmune response (24).

ACE2 may enhance the cytokine storm immune response through chemokine and pro-inflammatory cytokine expressions (25). These may activate adaptive immune cells (CD4 + and CD8 + T cells) and recruit innate immune cells (neutrophils, monocytes, macrophages, and natural killer cells) (26).

The long-term inflammatory persistence of Long COVID may be explained by the presence of residual SARS-CoV-2 antigens, resulting from low adaptive immune responses. Persistent activation of SARS-CoV-2-specific T cells has been observed at the beginning of infection and 6 months post-infection. Patients with Long COVID have a specific increase in CD4 + T and CD8 + T cells at the end of their recovery (27). According to Galán et al. (2022) (28), individuals with Long COVID have lasting cytotoxic persistence as indicated by high levels of CD8 + T and CD4 + T cells and PD-1 exhaustion markers on CD3 + . This may explain why this population develops a potent memory response against SARS-CoV-2.

The innate immune response is highly activated in patients with Long COVID, and this activation persists for 8 months after initial infection by SARS-CoV-2. This results in the increased expression of type I interferon (IFN-β) and type III interferon (IFN-β, -λ1) cytokines, T lymphocyte types CD38, CD86, CD14+, and CD16+, as well as monocytes. Immunological profiles of the patients revealed a chronic inflammatory response (29). Patients with Long COVID are likely to be in a state of autoimmunity conferred by a significant increase in interleukin (IL)-9-mediated Th9 cells. Autoinflammation and autoimmunity may also be maintained through IL-6 dysregulation (30).

Neuronal nicotinic acetylcholine receptors (nAChRs) are primarily found in the CNS and PNS. α7 nAChRs are found in the immune system of patients with COVID-19 and may persist long-term in various immune cells, such as macrophages, B cells, T cells, and dendritic cells. Activation of α7 nAChRs in the cholinergic anti-inflammatory pathway inhibits the production of pro-inflammatory cytokines (19). α7 nAChRs are present in neuronal and non-neuronal cells; a decrease in their levels would cause tissue damage and overproduction of cytokines. The Y674-R685 region of protein S can bind to α7 nAChRs and regulate ACE2. α7 nAChR expression ligands may affect SARS-CoV-2 infectivity and COVID-19 progression (31).

α7 nAChR deficiency causes immune and neuronal deficits. The SARS-CoV-2 spike protein (S12) facilitates viral cell entry. At a cellular level, S12 can suppress α7 nAChR. The S12 immunoreaction suggests a contribution to the onset of cardiovascular diseases independent of viral infection (23). In the ANS autoimmune imbalance, disparities autoantibodies at the G protein-coupled receptors contribute to the development of clinical and autonomic symptoms in patients with Long COVID (32, 33).

The α7 nAChR subtype is overexpressed in the hippocampus and is the most important mediator of the anti-inflammatory properties of the cholinergic system; dysregulation in this system could potentially cause the uncontrolled inflammatory response observed in COVID-19 (34). At the end of the inflammatory response, α7-nAChR is activated by acetylcholine, a neurotransmitter used by the parasympathetic nervous system (PSNS) (35).

After an inflammatory response, afferent signals are conducted through the vagus nerve (the primary nerve of the PSNS) to the nucleus tractus solitarius. Posteriorly, the vagus nerve is responsible for a reflex action called the cholinergic anti-inflammatory pathway(CAP) (36). This inflammatory reflex is dynamic, and immune responses can be influenced by the sympathetic nervous system (SNS). The SNS is connected to different central circuits and works as a protective neural system, performing tissue repair and global recovery through interactions between the immune system and the brain. The brain receives immune signals through cytokines and modulates immune system reactivity through the SNS and possibly the sympatho-adrenal and hypothalamic-pituitary-adrenal systems. During inflammation, cytokines are involved in nociceptor sensitisation in SNS fibres (37).

The ANS controls inflammation through the CAP (32). ANS and the immune system interact with invading pathogens. Acetylcholine and noradrenaline regulate cytokine release, and cholinergic signals from the efferent vagus nerve and α7 nAChR have anti-inflammatory functions. Intracellular signals interrupt NF-κB and activation of JAK2/STAT3 cascades, and inflammasome-mediated cholinergic signals using α7 nAChR stop the production of TNF, IL-1β, and other pro-inflammatory cytokines. The resolution of inflammation occurs through the synchronized action of protective mechanisms, lipoxins and eguasins, and increased activity of neutrophils and macrophages. The vagus nerve acts as an integrator during anti-inflammatory control (38).

Other mechanisms leading to Long COVID may include an association between immune-mediated vascular dysfunction, thromboembolism, and NS dysfunction. A relationship between the activation of the SNS and an increase in catecholamines accompanied by a cytokine storm and impairment mediated by ANS inflammation is likely to be established (39). Autonomic dysfunction in Long COVID can be caused by the direct viral action of SARS-CoV-2 or by the immune response impacting the ANS (40).

**Figure 1:**
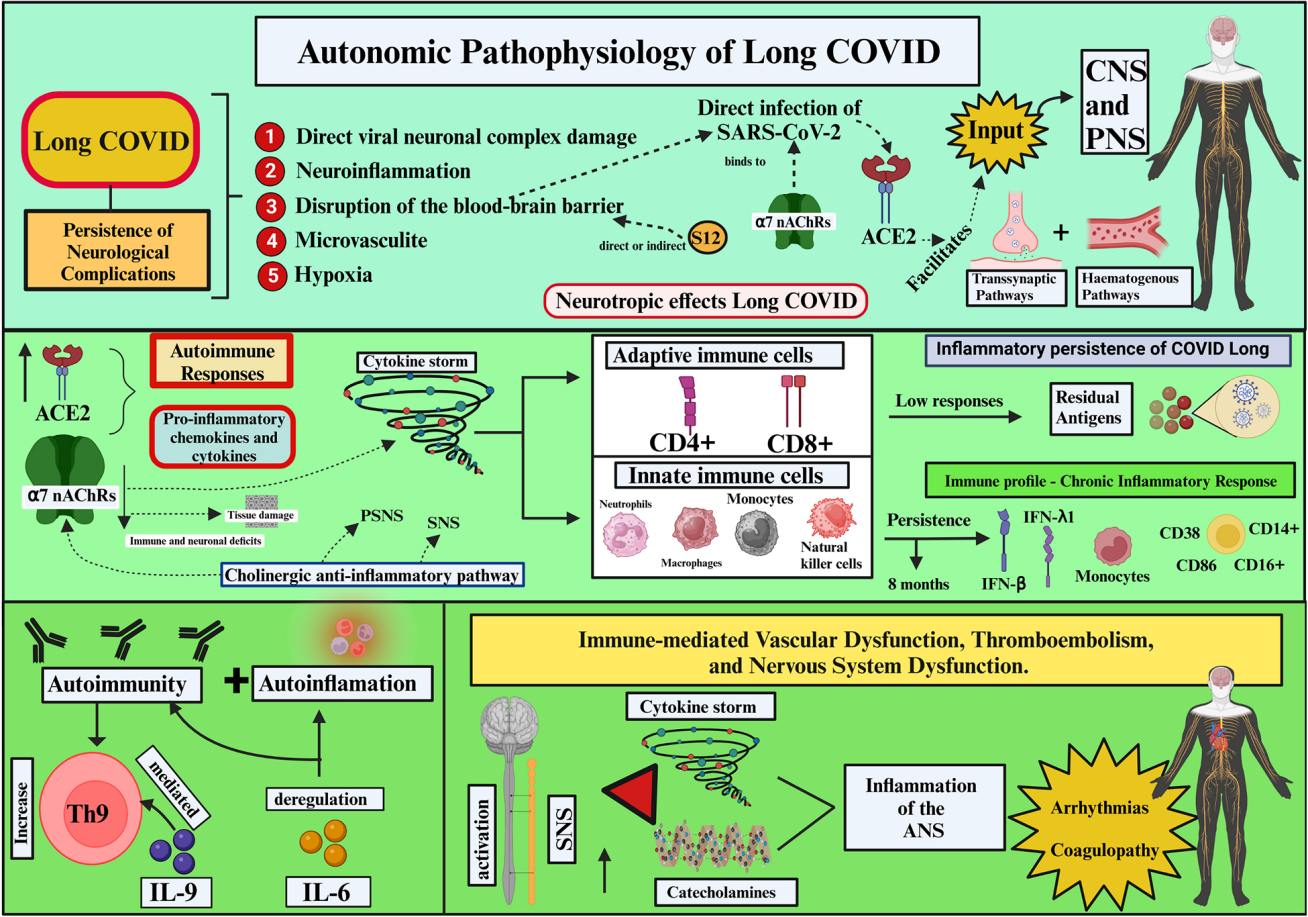
Autonomic Pathophysiology of Long COVID (Created with BioRender.com). SARS-CoV-2, severe acute respiratory syndrome coronavirus 2; ACE2, angiotensin-converting enzyme 2; S12, SARS-CoV-2 spike protein; α7 nAChRs, α7 nicotinic acetylcholine receptors; CNS, central nervous system; PNS, peripheral nervous system; PSNS, parasympathetic nervous system; SNS, sympathetic nervous system; CD4+, T lymphocyte CD4+; CD8+, T lymphocyte CD8+; type III interferon (IFN-β, -γ1), T lymphocyte types: CD38, CD86, CD14α, CD16α; IL-9, interleukin 9; IL-6, interleukin 6; ANS, autonomic nervous system.

Additionally, prolonged inflammatory persistence and cellular damage from fibrotic changes can reduce cell adhesion and lead to arrhythmias (palpitations) such as coagulopathies and postural orthostatic tachycardia syndrome (POTS) (41). According to Glynne et al. (2022) (42), patients with Long COVID may report improvements in symptoms, but symptoms of dysautonomia may persist (Figure 1).

## 3. HRV and inflammatory markers

In the meta-analysis performed by Williams et al. (2019), the link between HRV and inflammation was clarified. Higher HRV (all indices), particularly vagal HRV, was associated with lower levels of inflammation. Over time, the SDNN index proved to be the most consistent, while the high-frequency HRV (HF-HRV) was the strongest when examining frequency; these data support the involvement of the CAP. Examining the vagal index, both HF-HRV and RMSSD were related to inflammation. The inflammatory markers C-reactive protein (c-RP) and white blood cell (WBC) count exhibited negative associations with different levels of HRV (36).

According to Sotak (2022), inflammatory biochemical markers can be used to support and monitor infection diagnosis and treatment efficacy (43). Studies evaluating inflammatory markers and HRV in Long COVID are scarce, although Aeschbacher et al. (2017) (44) reported that the inflammatory parameters c-RP, leukocytes, and leukocyte subtypes may be associated with increased heart rate (HR) and decreased HRV (SDNN). These results suggest an association between inflammation and the ANS as well as increased cardiovascular morbidity and mortality. Autonomic dysfunction may put individuals with elevated inflammatory biomarkers at increased cardiovascular injury risk.

Elevated c-RP levels are associated with a chronic inflammatory state that functions as a peripheral marker in clinical research and may be associated with disease complications (45, 46). According to Pasini et al. (2021) (47), patients with Long COVID have high levels of c-RP and lactate dehydrogenase, resulting in cell damage and persistent inflammation.

HRV has the potential to aid in early inflammatory response detection and tracking. However, further HRV studies are required to study chronic inflammation states (48). The PSNS participates in inflammatory processes through HF-HRV and is inversely associated with IL-6, c-RP, and fibrinogen levels. This suggests that parasympathetic modulation of inflammation by the vagus nerve may act on specific inflammatory molecules. Similar inverse associations were observed between low frequency HRV (LF-HRV) and IL6 and c-RP (49).

The inverse association between HRV and the inflammatory markers IL-6 and c-RP in healthy individuals and those with cardiovascular disease (CVD) was reported by Haensel et al. (2008) (50). This indicated that HRV is associated with inflammation. The CAP driven by the vagus nerve is well described, and sympathetic and parasympathetic modulation substantially contribute to the modulation of cytokine production (51).

**Figure 2:**
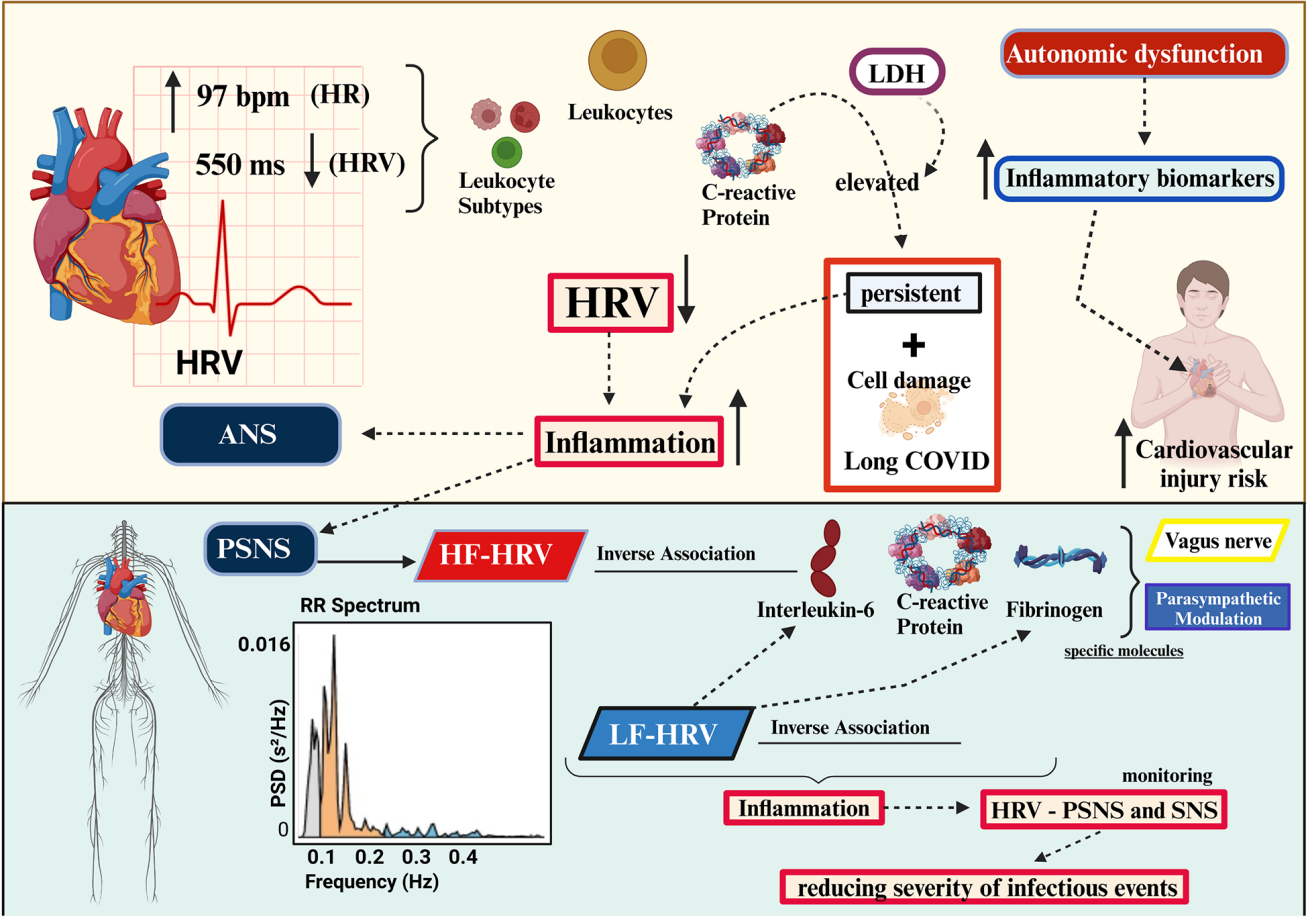
Association of heart rate variability and inflammatory markers (created with BioRender.com). HRV, heart rate variability; LDH, lactate dehydrogenase; ANS, autonomic nervous system; PNS, peripheral nervous system; PSNS, parasympathetic nervous system; SNS, sympathetic nervous system; HF-HRV, high-frequency HRV; LF-HRV, low-frequency HRV.

As in the study by Wegeberg et al. (2020) (52) pointing out the inflammation associated with HRV involving the SNS and PSNS. A cohort study (1,255 participants) conducted by Cooper et al. (2015) (53) found that LF-HRV was inversely associated with IL-6, c-RP, and fibrinogen levels, whereas HF-HRV was inversely associated with c-RP and fibrinogen. Vagal activity regulates systemic inflammation and prevents damage from excessive inflammatory responses (Figure 2). Implementing HRV monitoring associated with the monitoring of inflammatory markers could substantially contribute to reducing the severity of infectious events through early detection and reductions in mortality (54) (Figure 2).

## 4. Cardiovascular autonomic dysautonomia in long COVID

The negative and positive feedback mechanisms of the heart and vessels are regulated through the balance of inhibition and activation of sympathetic and parasympathetic afferent and efferent neurons of the Intrinsic Cardiac Nervous System. Cardiac neural regulation interacts reflexively to modulate heart rate and blood pressure. Stimulation of cardiac sympathetic afferents reflexively increase sympathetic efferents and inhibits efferent vagal cardiac fibers, generating an increase in HR and blood pressure (BP). While the parasympathetic cardiomotor function helps to reduce HR, blood pressure. Baroreceptor controls also reflect HR and BP fluctuations, wich convey responses for sympathetic and parasympathetic modulation (55, 56).

Long COVID may involve cardiovascular dysautonomia. POTS is the most prevalent type of CVD, defined as a persistent HR increase of at least 30 beats per minute when standing (upright). It may be associated with palpitations, chest pain, and exercise or orthostatic intolerance. POTS is more prevalent in women (80%) and can be precipitated by viral illnesses or serious infections (57, 58).

Eldokla et al. (2022) reported autonomic dysfunction in 332 individuals with Long COVID (59). The authors found a high prevalence of dysautonomia symptoms (76.7%) when using the Composite Autonomic Symptom Score 31 questionnaire (COMPASS-31). The high score (>16.4) found suggested that autonomic dysfunction was initially associated with a longer duration of Long COVID symptoms.

A high prevalence of dysautonomia (66%) was also reported by Larsen et al. (2022) (60) when using the COMPASS-31 questionnaire to evaluate 2,314 individuals with Long COVID; they observed moderate-to-severe autonomic scores. However, according to Hovaguimian (2023) (61), whether Long COVID is directly involved in autonomic impairment, causes POTS symptoms, or orthostatic intolerance through previously identified chronic disease mechanisms remains unclear.

Heart rate recovery provides a surrogate measure of autonomic health in patients with Long COVID. Long COVID POTS patients may benefit from conservative treatments to recovery HR and dysautonomia symptoms (62, 63), treatment may involve graded exercise, use of compression stockings, control of high fluid and sodium intake, and cognitive-behavioral therapy (57).

Dysautonomia was also reported by a cohort in a study by Zanin et al. (2023) performing a battery of autonomic tests on patients with Long COVID. Patients in this study had disabling symptoms and severe COVID-19. The authors showed more evident parasympathetic dysfunctions than sympathetic dysfunctions with fluctuating and polymorphic symptoms. Among the battery of tests, sympathetic and parasympathetic autonomic tests, neuropathy scores (Kale Score), and autonomic severity (evaluated using the Composite Autonomic Severity Score) were used. They found that 37.5% of patients had at least one abnormal test result, which was associated with mild autonomic failure in 83% and moderate autonomic failure in 16%. The cardiovascular system and sudomotor function were the most affected (64).

Dysautonomia can persist for almost a year due to symptom heterogeneity, and monitoring of cardiovascular autonomic dysfunction in patients with Long COVID is challenging. The impairment of these functions decreases the quality of life of patients, affecting their daily activities and hampering their ability to return to work (65).

The physical inactivity and at rest result in cardiovascular deconditioning and impair cardiovascular neural control (55, 66). HRV feedback training aims to increase vagal tone by metronomic breathing (5). Post-COVID-19 rehabilitation with an aerobic and resistance training program can provide important cardiorespiratory, cardiovascular, functional and autonomic responses (67).

## 5. Association of ANS and HRV

HRV measurement assesses the ANS with its interconnected sympathetic and parasympathetic branches (68). Heart rate and rhythm are considerably influenced by the ANS; parasympathetic activity decreases HR through the vagus nerve and sympathetic resolution by activating β-adrenergic receptors. HRV can be measured using either linear or nonlinear methods. High HRV or one that is reduced are linked to sympathetic (“escape”) and parasympathetic activation (“recovery”), respectively (69).

In general, autonomic imbalances refer to the inappropriate ability of the autonomic system to respond physiologically to stimuli, either by increased or decreased modulation of one of the two branches. Inappropriate increased or decreased of vagal or sympathetic modulation can be detrimental (7, 70, 71), and generate clinical implications (72). The dysautonomia can be found due to prolonged bed rest, so early initiation of rehabilitation can combat its symptoms (5). Physically inactive people (mild to moderate infection) have greater autonomic dysfunctions (increased sympathetic activity—LF) compared to physically active controls (increased parasympathetic activity—HF) (73).

With HRV reduction, neuroautonomic disconnections or a decrease in baroreflex sensitivity can be observed. HRV reduction can be estimated by reductions in time-related factors (DNN, RMSSD, and pNN50) or frequency (LF/HF ratio reductions or LF increases). Infectious diseases can reduce HRV, and ANS imbalances can reduce the vagal tone and compromise physiology by uncoupling distinct mechanisms (74).

A high HRV indicates a sympathovagal balance and good ANS adaptability. A reduced HRV may indicate abnormal ANS control (75). Activation of the inflammatory reflex has been proposed to interfere with vagal modulation of the HR as inflammation in the acute and chronic phases modifies cardiac function through changes in HRV (76).

**Figure 3:**
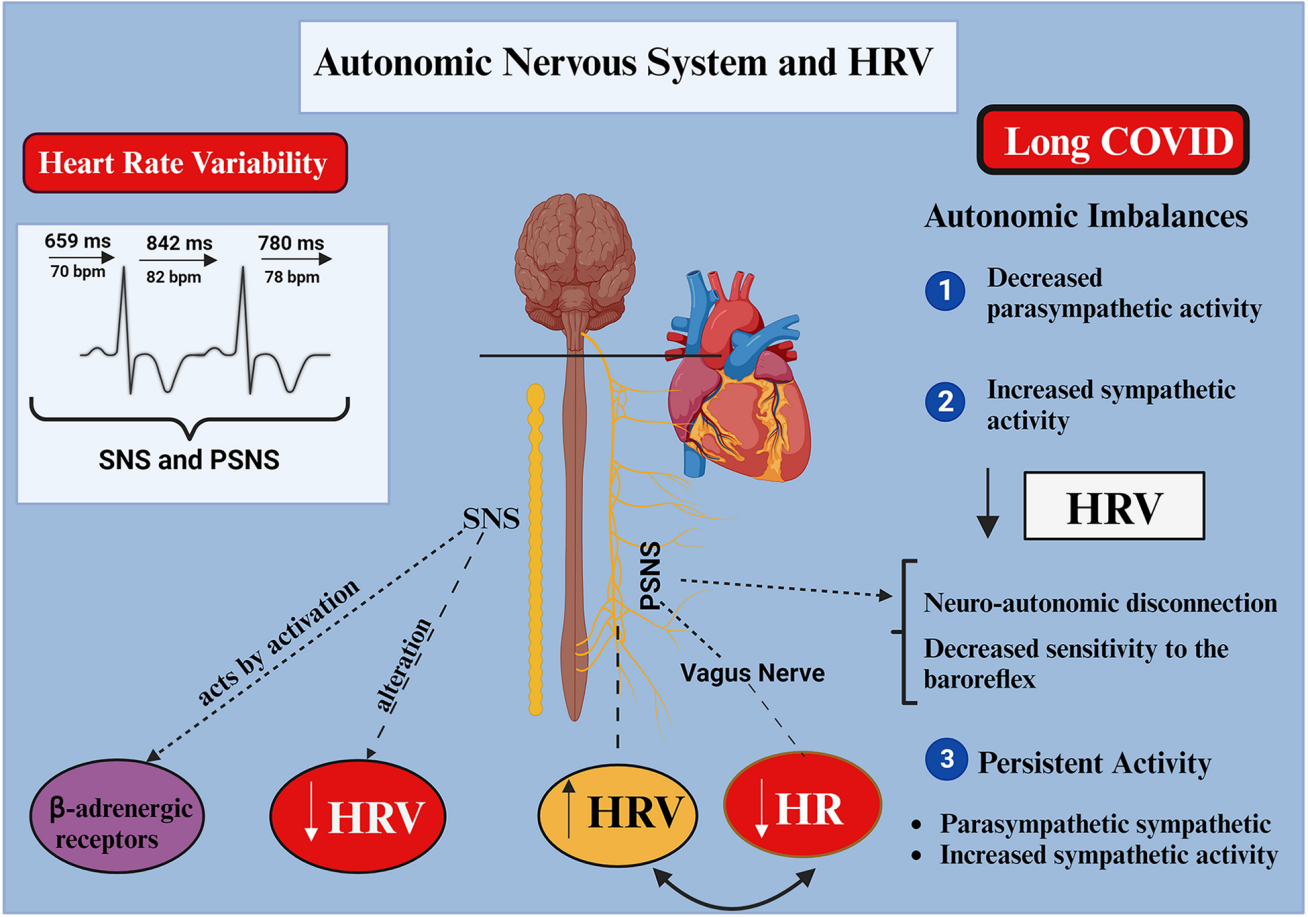
Autonomic nervous system and heart rate variability in Long COVID (created with BioRender.com). SNS, sympathetic nervous system; PSNS, parasympathetic nervous system; HRV, heart rate variability; HR, heart rate.

The vagus nerve contributes to inflammation control through its afferent and efferent pathways, playing a dual anti-inflammatory role. Low vagal tone, visualised by HRV, is a marker of sympathovagal balance (77). A wide range of symptoms are observed in the population with Long COVID. Therefore, autonomic testing, including checking blood pressure, heart rate, and HRV by long-term Holter monitoring (24 h) or short-term heart rate monitoring (5–15 min) is imperative. These tests can be adopted in clinical practice (65, 78, 79) (Figure 3).

## 6. Heart rate variability in long COVID

HRV is considered an indirect biomarker of cardiac autonomic control. A reduced HRV can be observed in cardiac and non-cardiac patients and is related to a worse prognosis (80). During viral infections, changes in long-term autonomic control and individual responses to HRV can be observed (81).

HRV can indicate cardiovascular dysautonomia in individuals suffering from Long COVID. These patients may have reduced HRV compared with healthy individuals. Data predicting autonomic dysfunction in patients that have recovered from SARS-CoV-2 infection are limited (82, 83). Dysautonomia was also reported by Liviero et al. (2023) in paucisymptomatic patients that had recovered from acute COVID-19. With analysis of HRV frequency and time, persistent sympathetic activity (increase in LF and LF/HF) and decreases in vagal activity due to decreases in HF were observed, in addition to reductions in SDNN and RMSSD. Patients in the acute phase of COVID-19 with autonomic dysregulation can progress to Long COVID; HRV works as a marker of worsening clinical condition in the long term (84).

To verify dysautonomia, Acanfora et al. (2022) examined HRV in 30 individuals with Long COVID and found impaired vagal activity and sympathovagal imbalances. The authors found lower SDNN, SDANN, and SDNNi values in patients with Long COVID. Examining frequency, patients presented lower total power for VLF and HF components. When stratifying patients with Long COVID and controls, the SDNN parameter was lower in Long COVID, whereas the LF/HF ratio, D-dimer, NT-Pro-BNP, and IL-6 were significantly higher (85).

Vagal dysfunction and sympathovagal alterations identified through HRV can be visualised by changes in HF components and LF/HF ratio. Persistent parasympathetic activity has been reported in individuals with Long COVID by Menezes Júnior et al. (2022). Patients with Long COVID showed lower HF values than healthy individuals but had LF increases. The authors discussed that increases in parasympathetic activity (for example, in RMSSD) might be related to symptoms of Long COVID and inflammatory marker increases (brain natriuretic peptide, D-dimer, and c-RP) (86).

An increase in parasympathetic tone initially overcome by an increase in sympathetic tone was visualised in 60 patients with Long COVID (>12 weeks post-COVID) in a study by Asarcikli et al. (2022). In comparison to healthy controls, the Long COVID group showed a significant increase in SDNN, RMSSD, and HF when examined using by the VFC in the domain of time and frequency. Prolonged parasympathetic activity may be responsible for the varied symptoms seen in Long COVID (87). However, it is noteworthy that in this study the authors excluded asymptomatic patients or with severe COVID-19 infection, as well as patients with depression, renal failure, morbid obesity diabetes, obstructive sleep apnoea and overt cardiovascular diseases (all known to have a reduced vagal tone), such exclusion criteria were not considered, which may generate selection bias since their result can only be applied to a small category of Long COVID patients, but ultimately not to all patients with Long COVID.

Shah et al. (2022) analysed cardiovascular dysautonomia in 92 patients that had recovered from COVID-19 by examining HRV over time. They found that patients had significantly reduced HRV and higher levels of inflammatory markers than controls. Reduced HRV negatively correlated with RMSSD in relation to the c-RP and IL-6 markers. The HRV time domain (RMSSD) was the most relevant measure for short-term ANS analysis (83).

Lampsas et al. (2022) evaluated patients with Long COVID who had autonomic dysfunction and were hospitalised (88). These patients experienced decreases in overall SDNN-HRV when evaluated with 24-hour ambulatory Holter analysis. The SDNN improved within 6 months of hospitalisation, corroborating the association between autonomic dysfunction and Long COVID. In contrast, Kurtoğlu et al. (2022) examined 50 patients with Long COVID with cardiovascular autonomic dysfunction who were not hospitalised (89). They found that cardiovascular autonomic dysfunction was associated with a decrease in RMSSD parameters, pNN50, HF, and LF, as well as a decrease in signal complexity seen by approximate and sample entropies.

Many authors have reported a loss of autonomic function due to SARS-CoV-2 infection; Freire et al. (2023) (90) reported that seven immunised (full immunisation) young adult patients with Long COVID experienced restoration of their autonomic function over approximately 5 months after mild to moderate infection. The improvement in autonomic function should be further investigated to follow the progression of SARS-CoV-2 infection and enhance the understanding of its long-term effects on the ANS.

## 7. Importance of HRV for long COVID

For better long-term management, physicians should be aware that patients with mild to severe symptoms of COVID-19, as well as asymptomatic people, can develop symptoms of Long COVID regardless of their health status (91). Long COVID symptoms can last for weeks, months, or even years after the acute phase of the illness. Melatonin is a neuroprotective drug that has shown promise in aiding Long COVID recovery as it helps combat cytokine reactions and advancing symptom severity, helps prevent neurological disorders, and controls cognitive deterioration (“brain fog”) (92).

HRV is influenced by the circadian rhythm; it can decrease throughout the day with higher morning values, with RMSSD and SDNN being higher during the day. The cardiovagal response to stress generates higher HRV at rest, and cardiovagal control is essential to reduce chronic disease symptoms (93). Patients with Long COVID often have insomnia and circadian rhythm problems (94), and treatment with melatonin can substantially combat these deficits in addition to decreasing dysautonomia and increasing HRV (95).

Different HRV responses are observed in patients with Long COVID. Karakayali et al. (2023) examined patients with Long COVID (mild to moderate COVID-19) that were symptomatic or asymptomatic; symptomatic patients had higher parasympathetic tone (RMSSD, SDNN, SD1, and SD2). The parasympathetic system increases HRV, whereas vagal dysfunction reduces it (96).

HRV monitoring results in better stratification in patients with Long COVID. HRV can be used to monitor Long COVID (symptomatic), post-COVID-19 (asymptomatic), and uncertain Long COVID (unclear) individuals (97). Long COVID can range in severity from mild to severely debilitating (98). HRV monitoring in these groups should identify early diagnosis of autonomic changes, predict the severity of Long COVID, limit disease progression, improve clinical outcomes, and lead to new therapeutic strategies.

Analyses of complex and simple HRV markers over time and frequency have been performed to detect early changes in autonomic involvement. Patients with low HRV are at risk of arrhythmia and sudden death (6); furthermore, reduced HRV can be considered predictive of malignant arrhythmias (99).

Different clinical groups of patients with Long COVID were analysed by Marques et al. (2022) using HRV measurement with linear and non-linear methods. They found increased sympathetic activity (LF, LF/HF) at rest and reduced parasympathetic tone (RMSSD, SDNN, SD1, and HF). These alterations can lead to increases in heart rate and blood pressure, predisposing patients to cardiovascular complications, chronic diseases, and sudden death (78).

Owing to its affordability, HRV monitoring can be adopted into evaluation protocols to monitor therapies and the improvement of patients with Long COVID who have autonomic dysfunction (100). Severely ill patients who do not show improvements in long-term HRV require a longer follow-up period to eliminate the virus. HRV markers can be associated with clinical disease severity, acting as non-invasive monitoring resources and predictors of clinical outcomes (101).

HRV is the most appropriate tool for diagnosing patients with Long COVID who have cardiovascular autonomic dysfunction as it generates a quantitative score independent of cognitive function. In addition, HRV can be used as a predictor of inflammatory and autonomous states using diagnostic and predictive methods for cardiovascular function (102). This provides further information on the vagal anti-inflammatory role (53), indicators of cardiovascular health, and the mortality prognosis for the Long COVID population (103, 104).

Neurocognitive “brain fog” symptoms can remain elevated up to 2 years after SARS-CoV-2 infection, as described in a cohort of a retrospective study of nearly 1.3 million patients (105). Autonomic dysfunction can also occur with the presence of persistent dyspnoea, and 60% of patients with Long COVID progress without improvement (106). Monitoring HRV from the initial clinical diagnosis to full recovery may be fundamental for understanding the autonomic balance of patients with Long COVID.

## 8. Future research

Future studies are needed to clarify the changes in cardiovascular autonomic modulation seen in the Long COVID population. Although proposals for pathophysiological mechanisms have been discussed, the data in the literature remain unclear. It is necessary to identify whether there is a relationship between inflammatory markers and an autonomic origin related to the cardiovascular system.

## 9. Conclusions

Dysautonomia in patients with Long COVID is multifactorial, and many of the autonomic symptoms can be understood by vagal dysfunction; the parasympathetic and sympathetic interactions involved still need to be extensively studied. Understanding cardiovascular autonomic dysfunction and the importance of association with inflammatory biomarkers in patients with Long COVID can contribute to more accurate diagnoses, better knowledge of the clinical presentation of the ANS pathway when reacting to an infectious disease, and better prognoses. Future studies are needed to understand how the recovery of the autonomic pathway occurs in patients with Long COVID.
